# Consumption of fruits and vegetables among adolescents in Arab Countries: a systematic review

**DOI:** 10.1186/s12966-022-01398-7

**Published:** 2023-01-09

**Authors:** Widad Zeidan, Haneen Taweel, Aisha Shalash, Abdullatif Husseini

**Affiliations:** Institute of Community and Public Health, Birzeit University, Birzeit, Palestine

**Keywords:** Fruits, Vegetables, Consumption, Adolescents, Arab countries

## Abstract

**Background:**

Adolescents’ consumption of fruits and vegetables is inadequate in most Arab countries, leading to a higher risk of poor health outcomes. This systematic review evaluates fruits and vegetables intake among adolescents in Arab countries, the proportion of adolescents meeting the dietary guidelines in these countries, and the dietary assessment tools used to assess fruits and vegetables intake.

**Methods:**

Four databases were searched, MEDLINE, PUBMED, EMBASE, and Web of Science. Studies were eligible if they reported fruit or vegetable consumption among adolescents aged 10 to 19 in 22 Arab countries. The risk of bias in the included studies was assessed by two reviewers independently using the risk of bias tool developed by Hoy et al. Data were extracted and synthesized into three categories; frequency of fruits and vegetables consumption, mean fruits and vegetables consumption, and percentage of adolescents meeting fruits and vegetables consumption recommendations.

**Results:**

The review included 44 articles utilizing 41 cross-sectional studies. Most studies were school-based, and data was collected from both males and females using self-administered questionnaires. Of those, validated questionnaires were used in 28 studies. According to the World Health Organization recommendation, most studies defined five fruits and vegetables servings as the adequacy cutoff point; other definitions were used in some studies. The reported mean consumption ranged between 6.1 times per week and 4.5 servings of fruits and vegetables per day. The proportion of those who met the recommendations of eating five servings per day ranged between 10 and 29%. Fruits were shown to have a lower daily intake than vegetables (4.2 to 53.7% for fruits and 7.8 to 66.3% for vegetables).

**Discussion:**

This review indicated inadequate fruits and vegetables consumption among adolescents in Arab countries and highlighted an increased risk of non-communicable diseases and malnutrition prevalence. A limitation was the incomparability of available data between countries. Further in-depth research on the core reasons behind adolescents’ inadequacy in fruits and vegetables consumption is recommended.

**Supplementary Information:**

The online version contains supplementary material available at 10.1186/s12966-022-01398-7.

## Background

According to the World Health Organization (WHO), daily consumption of fruits and vegetables (FAV) could decrease the risk for several non-communicable diseases (NCDs). Furthermore, eating fruits and vegetables as part of a healthy diet low in sugar, salt, and fat is thought to help prevent weight gain and obesity, which is an independent risk factor for NCDs [1]. Consuming the appropriate amount of FAV throughout adolescence has been linked to improved future health outcomes, including a lower risk of various non-communicable diseases such as strokes, coronary heart disease, and cancer [2].

Increased fruits and vegetables intake during adolescence offers both long-term protective benefits and short-term benefits such as improved school performance [3]. Even though FAV consumption is associated with a lower risk of chronic diseases, consumption among adolescents remains insufficient [4]. For example, research in Europe and North America found that most adolescents did not consume the recommended amount of FAV per day [2]. Furthermore, data reveal that FAV consumption among adolescents is low in many Arab countries. For example, according to statistics from the Global School-based Student Health Survey (GSHS), consumption of fruits and vegetables among adolescents aged 13–15 in the Eastern Mediterranean Region (EMR) is low, ranging from 12.6% in Libya to 38.1% in Djibouti [5].

Fruits and vegetables preferences, racial/ethnic disparities, family rules and support, gender, age, FAV availability and/or accessibility, willingness to consume healthy foods, and household characteristics are all factors that influence FAV consumption among adolescents. For example, the family has a large influence on FAV intake because it shapes adolescents’ eating beliefs and is the main food provider in a household. As a result, adolescents are more likely to consume nutritious or unhealthy foods based on what is available at home and how they are influenced by their family’s eating patterns [6, 7]. Furthermore, socioeconomic status [8], family income, education of the head of the family, smoking [9], peer influence [10], parents’ eating habits, social setting, television watching, dietary trends, and more time spent away from home [6] all influence FAV consumption during adolescence. Due to restricted options and time, adolescents who spend time away from home, whether at school or with friends, are more likely to eat convenient pre-packaged meals and snacks, which affects their eating habits, including their FAV consumption [6].

Although failure to reach the daily FAV requirement puts adolescents at risk of acquiring chronic diseases [11], it is necessary to focus on the adolescent age group when addressing eating habits and health for several important reasons. First, they must establish good eating habits, as evidence has shown that behaviors formed at this age are more likely to be maintained into adulthood [2, 4]. Furthermore, rapid growth happens during this age period, increasing the nutrient requirements among adolescents to meet the needs for growth and development [2]. Finally, because adolescence is a vital era, it is beneficial to use it to modify adolescents’ food habits and consumption patterns, including fruits and vegetables consumption [12]. It is essential to assess the present consumption patterns of fruits and vegetables among adolescents to execute successful interventions and establish practical policies that would help improve fruits and vegetables consumption [13]. As a result, performing a systematic review will provide valuable information into adolescent FAV consumption in the Arab region, as well as changes in consumption throughout this age group in prior years. This understanding will be critical in developing appropriate policies to boost FAV consumption as well as the implementation mechanisms of programs aimed at increasing FAV consumption among adolescents.

To effectively compare FAV consumption among specific populations or groups utilizing review studies, evaluation methodologies must be standardized as each method has advantages and disadvantages [14]. Examining current dietary intake measuring methods in Arab countries may thus inform future standardization of FAV consumption procedures. Food Frequency Questionnaires, 24-hour recalls [15], food diaries [16], and self-administered questionnaires are routinely used to assess FAV consumption [14]. However, the inconsistency of dietary intake measuring methodologies in research publications makes drawing inferences and comparing data from different papers more difficult [17]. When various approaches were examined, it was discovered that some overestimated intake compared to others [16], and 24-hour recollections provided a more significant result in measuring FAV consumption [17].

The objective of this review is to estimate FAV consumption among adolescents in Arab countries while also investigating the various methodologies used to quantify FAV consumption, the proportion of adolescents meeting the recommendations, and the variation among countries. This review significantly contributes to the existing literature by supplying data on the amount of fruits and vegetables consumed by adolescents in various Arab countries, as well as consumption data itemized according to the various dietary outcome methodologies used to collect and present such data. The consumption data includes the frequency of FAV consumption, mean FAV consumption, and percentage of adolescents meeting the recommendations.

## Materials and methods

The protocol was registered in the International Prospective Register of Systematic Reviews (PROSPERO) and can be found in the PROSPERO record (ID: CRD42020202818) [18]. The planning and implementation of this review followed the guidelines of the Preferred Reporting Items for Systematic Reviews and Meta-Analyses (PRISMA) system [19] (Additional file 1).

### Search strategy

We systematically searched the literature for all relevant articles in four databases: MEDLINE, PUBMED, EMBASE, and Web of Science, in August 2020. A grey literature search was not performed as we were more interested in including only peer-reviewed articles.

The following combinations of keywords were employed using the advanced search function in each of the different databases: (Fruit* OR vegetable*) AND (Intake OR consumption OR diet OR dietary OR eating OR nutrition) AND (Adolescent* OR child* OR student* OR teen*) AND (Egypt OR Iraq OR Jordan OR Lebanon OR Saudi Arabia OR Syria OR Yemen OR Libya OR Sudan OR Tunisia OR Kuwait OR Algeria OR Bahrain OR Qatar OR Oman OR United Arab Emirates OR UAE OR Morocco OR Mauritania OR Somalia OR Palestin* OR West Bank OR Gaza OR Djibouti OR Comoros OR Arab).

### Inclusion and exclusion criteria

Studies were eligible if they reported fruit or vegetable consumption among adolescents aged 10 to 19 years living in 22 Arab countries. Eligible studies included observational studies (cross-sectional, cohort, and case-control) and experimental (Intervention studies) and targeted the general population (population-based studies). Studies were excluded if they were systematic reviews, special-population studies (participants defined with a disease or a disorder), 18 years and older- population studies, or if the article was neither in English nor Arabic.

### Study selection

Title and abstract screening were completed by two independent reviewers; differences were addressed and resolved by consensus. Similarly, full-text screening was undertaken by two separate reviewers; disagreements were resolved through discussion between both reviewers or by a third reviewer. The Covidence platform was used to complete both title and abstract and full-text screening [20].

### Data extraction quality assessment

Data were extracted and recorded independently by two reviewers using Excel and presented using tables. Reviewers recorded missing data as not available. Disagreements were resolved through consensus, and a third reviewer was referred for any unresolved conflicts. Extracted data included publication year, data collection year, country/countries of participants’ residence, outcomes of interest, study design, sample size, demographics and participants’ characteristics, dietary assessment methodology, dietary measurement, the validity of the dietary measure, reliability of the dietary measure, dietary variables, measures of dietary variables, statistical analysis method, and variables adjusted for, and measures of dietary outcome.

Both reviewers independently assessed the risk of bias in the included studies using the risk of bias tool developed by Hoy et al. [21]. The tool was simple to use and worked well with cross-sectional studies. When specifics were absent in an article, data were taken from the references. When a bias risk criterion was uncertain, it received a high-risk rating.

### Data synthesis

Data on fruits and vegetables consumption were classified into three major categories: frequency of consumption, mean consumption, and percentage of adolescents meeting the recommendations. First, the reported intake in grams was converted to servings to calculate the consumed servings of fruits and vegetables by dividing consumed grams over 80 g [3]. Second, when the percentage of adolescents meeting the recommendation was unavailable, the number of those who met the recommendations was divided by the number of participants. Third, the reported number was converted to servings in studies where FAV intake was only reported as calories by dividing calories from fruits and vegetables over 60 and 25 cal, respectively [22]. Fourth, cup consumption was converted to servings by multiplying by 2 [22]. Weekly consumption was converted to approximate daily by dividing by 7. Finally, adequacy was defined as consuming five servings of fruits and vegetables per day, and the data were extracted and synthesized separately in studies using other guidelines.

## Results

### Search results

**Fig. 1:**
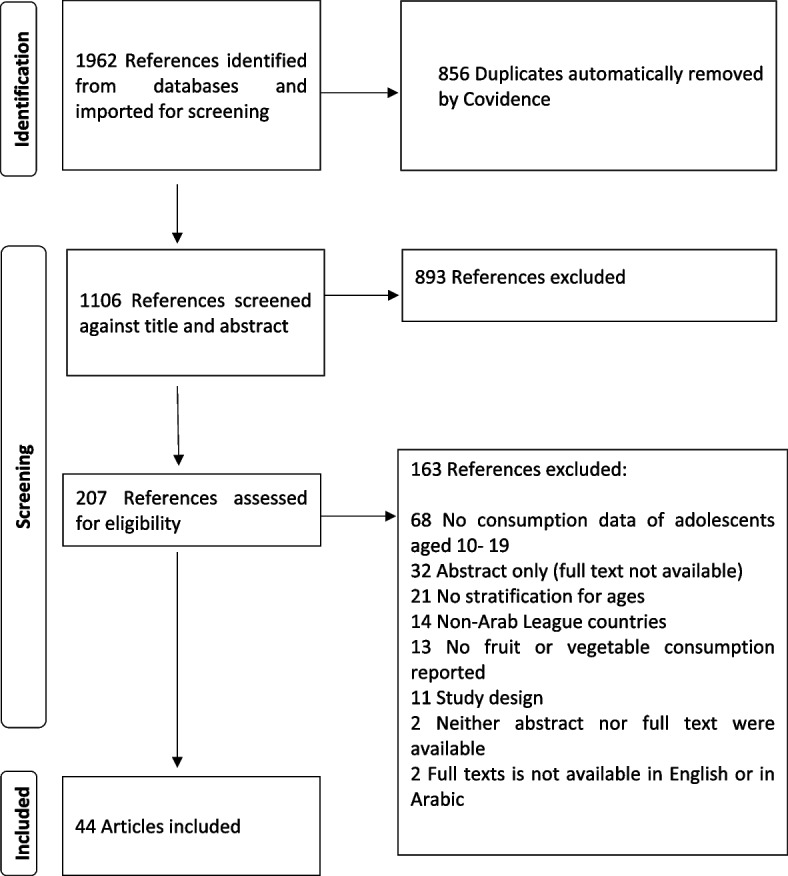
Flow chart for study retrieval and identification

A total of 1962 references were identified through our database search, and 207 studies were included for full-text screening. Forty-four articles were included in the review. These articles reported data from 41 independent studies. Figure 1 is the PRISMA flow chart of included articles.

Some studies were excluded despite seeming to meet inclusion criteria. One study was excluded as FAV consumption was only reported regarding the proportion of adolescents consuming FAV at each meal [23]. Three quasi-experimental studies [24–26] and a case-control study [27] were excluded because participants were of specific characteristics or reported consumption was of a given intervention or control. Another study was excluded because FAV consumption was reported only by categories of physical activity [28].

The majority of the included studies assessed dietary habits or behaviors. The data for the studies included in this review were collected between 1999 and 2018. Data was collected in sixteen studies between 1999 and 2009 and in seventeen studies between 2010 and 2018. In ten studies, the year of the collection was not mentioned. The sample sizes ranged from 120 to 164,771 participants, and the majority of the studies were cross-sectional studies conducted in schools. Twelve studies were carried out in Saudi Arabia, seven in Morocco, six in Jordan, five in Palestine, four in Lebanon, three each in Oman, Syria, the United Arab Emirates (UAE), Tunisia, and Egypt, and two each in Bahrain, Kuwait, Yemen, Sudan, Libya, and Djibouti, and one each in Iraq, Mauritania, and Algeria. A summary of study characteristics can be found in Table 1.

**Table 1:** Summary of characteristics of studies

Characteristics	*Total*
Publication year	
2005 or before	1 (2%)
2006-2010	5 (11%)
2011-2015	18 (40%)
2016-2020	20 (46%)
End of data collection	
1999- 2004	5 (12%)
2005- 2009	11 (26%)
2010 - 2013	7 (16%)
2014- 2018	10 (23%)
Not mentioned	10 (23%)
Country^a^	
Saudi Arabia	12 (30%)
Morocco	7 (18%)
Jordan	6 (15%)
Palestine	5 (13%)
Lebanon	4 (10%)
Oman, Syria, UAE, Tunisia, Egypt	3 (8%)
Bahrain, Kuwait, Yemen, Sudan, Libya, Djibouti	2 (5%)
Iraq, Mauritania, Algeria	1 (3%)
Number of participants	
Less than 500	8 (19%)
500-1000	13 (30%)
1001- 2000	8 (19%)
2001-5000	7 (16%)
5001-10,000	3 (7%)
12,575	1 (2%)
24,220	1 (2%)
26,328 (multiple countries)	1 (2%)
164,771 (multiple countries)	1 (2%)
Sex	
Studies with males and females	38 (88%)
Male-only study	3 (7%)
Female only study	2 (5%)
Methodology	
Food frequency questions	40 (93%)
24- hour recall	2 (5%)
FFQ+ 24-hour recall	1 (2%)

^a^Studies on multiple countries counted for each country

### Participants demographics

The majority of the included studies were school-based and included both males and females. Three studies only looked at males, while the other two looked only at females. The ages ranged primarily from 12 to 18 years. Additional file 2 contains an overview of the included studies, and Table 1 presents a summary of them.

### Dietary assessment methodologies

Except for one study, all data were reported using a questionnaire. Twenty-seven of those questionnaires were self-administered. Five of the studies used the GSHS questionnaire, and two used the Arab Teens Lifestyle Study questionnaire [29, 30]. One of these studies used an online questionnaire to collect data. Assessment tools in the included studies primarily consisted of food frequency questions. In addition, a 24-hour recall questionnaire was used in three studies, along with a Food Frequency Questionnaire (FFQ) in one. Only two studies did not report data on vegetable consumption, while all assessed fruit consumption. In addition to fruits and vegetables, thirty-five studies investigated the consumption of other food groups. Eight studies compared unhealthy food consumption with healthy food consumption. In addition, two studies inquired about fruit juice as a separate item.

The majority of studies collected frequency data; fourteen studies collected frequency weekly, and twelve studies collected frequency daily. Two of these studies measured consumption quantities. In comparison, nine studies did not report the type of assessed dietary measurement (quantity, frequency, etc.). Eleven studies provided a reference period: five from the previous thirty days, four from the previous week, and two from the previous day. Most studies did not include data on the amount of fruits and vegetables consumed.

Overall, ten studies used a reliable questionnaire, and twenty-eight used a validated questionnaire, eight of which used the validated GSHS questionnaire. In nine studies, the validity of the questionnaire was not reported. Backward translation and questionnaire piloting were reported in five studies, four of which both were implemented. The WHO-recommended five servings of fruits and vegetables [31] were used as the adequacy cutoff point in most studies reporting the percentage of adolescents meeting recommendations. Nonetheless, other cutoff points were used in some studies. Two studies, for example, defined vegetable adequacy as three or more servings per day, and one of those studies defined fruit adequacy as two or more servings per day. Furthermore, three studies established their cutoff points as four servings of fruits and a comparable cutoff point for vegetables. Additional file 3 contains data on dietary assessment methodologies; validity and reliability information are reported as available in the included studies.

### Risk of bias assessment

No study ranked the high risk of bias on all criteria. However, more than half of the studies (*n* = 28) had an increased risk concerning their target population as it was not a clear representation of the national population. On the other hand, the sampling frame was classified as high risk in only four studies as it closely represented the target population in most studies. Moreover, six studies had a bias risk in sample selection. However, the non-response rates were often unclear (*n* = 21).

Except for one study, data was collected directly from the subjects in all studies, representing a low risk. Similarly, the case definition was acceptable in all but one study. Regarding the data collection instruments, most of the studies (*n* = 28) showed using valid and reliable questionnaires, while the rest (*n* = 16) had neither reliability nor validity. In all studies, the same mode of assessment was used for all participants. The reference period was unclear or inappropriate in 21 studies. Furthermore, the numerator and denominator for the dietary outcomes were appropriate in all studies. The risk of bias is provided in Additional file 4.

### Dietary outcomes

Regarding fruits and vegetables consumption, data was presented in means, percentage of students meeting the recommendations, and frequencies. The mean was reported among nine countries and ranged between 6.1 times per week and 4.5 servings per day (Table 2). Adolescents’ mean fruits and vegetables intake did not meet five servings per day in any country.

**Table 2:** Mean consumption

Study	Country	Mean Consumption ± SD	Units	Approx. Mean consumption/day	Unit/ Day
Ali et al. (2013) [32]	UAE	Vegetable: M: 0.8, F: 0.5 Fruit: M: 0.4, F: 0.6 SD not reported	cups/day	Vegetable: M: 1.6, F: 1 Fruit: M: 0.8, F: 1.2	Servings/day
Abudayya et. Al (2009) [33], Abudayya et. Al (2011) [34]	Palestine	FAV: 8.5 = 1.2 SD not reported	Frequency*/ week	FAV: 1.2	Frequency*/ day
Abu-Mweis et al. (2014) [35]	Jordan	Vegetables: 4.5 ± 2.1 Fruits:4.2 ± 2.2	Frequency*/ week frequency*/ week	Vegetables: 0.6 Fruits: 0.6	Frequency*/ day
Al-Hazzaa et al. (2011) [36]	Saudi Arabia	Vegetables: M: 3.71 ± 2.4, F: 3.63 ± 2.4 Fruits: M: 3.27 ± 2.3, F: 2.52 ± 2.1	Frequency*/ week	Vegetables: M: 0.5, F: 0.5 Fruits: M: 0.5, F: 0.4	Frequency*/ day
Al-Hazzaa et al. (2013) [37]	Saudi Arabia	Vegetables: 3.69 ± 2.4 Fruit: 2.85 ± 2.2	Frequency*/week	Vegetables: 0.5 Fruit: 0.4	Frequency*/ day
Allafi et al. (2014) [38]	Kuwait	Vegetables: M: 3.8, F:3.5 Fruits: M: 3.4, F: 2.8 SD not reported	Frequency*/week	Vegetables: M: 0.5, F:0.5 Fruits: M: 0.5, F: 0.4	Frequency*/ day
Al-Sagarat et al. (2017) [39]	Jordan	Vegetables: 3.31 ± 1.51 Fruits: 2.92 ± 1.28	Frequency*/ day	Vegetables: 3.31 Fruits: 2.92	Frequency*/ day
Aounallah-Skhiri et al. (2011) [40]	Tunisia	Fruit: 1.5 SD not reported	Calculated in servings/ day	Fruit: 1.5	Servings/day
Azekour et al. (2019) [41]	Morocco	Age group (10–12, ≥ 13) Vegetable: 10–12: 150.17 ± 37.09 Fruit: 10–12: 187.61 ± 66.82 Vegetable: ≥ 13: 161.33 ± 39.03 Fruit: ≥ 13: 198.55 ± 64.48	g/day	Age group (10–12, ≥ 13) Vegetable: 10–12: 1.9 Fruit: 10–12: 2.3 Vegetable: ≥ 13: 2 Fruit: ≥ 13: 2.5	servings/ day
Chacar et al. (2011) [42]	Lebanon	Salad: 3.66 ± 2.25 avg.: 3–4 times weekly Fruits: 5.67 ± 2.69 avg.: 4–5 times/week	Frequency*/ week	Salad: 0.5 Fruits: 0.8	Frequency*/ day
Collison et al. (2010) [43]	Saudi Arabia	Vegetable: 5.95 Fruit: 10.55 Fruit Juice: 3.85 SD not reported	servings/ week	Vegetable: 0.85 Fruit: 1.5 Fruit Juice: 0.6	Servings/ day
Ghrayeb et al. (2014) [44]	Palestine	Vegetable: 3.1 ± 1.5 Fruit: 3.2 ± 1.6	Servings/ day	Vegetable: 3.1 Fruit: 3.2	Servings/ day
Mikki et al. (2010) [45]	Palestine	FAV: M: 6.5, F: 6.1 SD not reported	Frequency*/ week	FAV: M: 0.9, F: 0.9	Frequency*/ day
Musaiger et al. (2014) [46]	Iraq	Vegetable: M: 5.3 ± 1.9, F:5.9 ± 1.71 Fruit: M: 4.5 ± 1.9, F: 5.3 ± 2.0	Frequency*/ week	Vegetable: M: 0.8, F:0.8 Fruit: M:0.6, F: 0.8	Frequency*/ day

*Frequency: Number of times

Fruit consumption ranged between 2.5 times per week to 2.9 servings per day. Vegetable consumption ranged between 3.3 times per week and two servings per day. Fifteen countries reported consuming five servings in twenty-three studies (Table 3). The proportion of adolescents meeting the recommended consumption of fruits and vegetables ranged from 5.4 to 40.4%.

**Table 3:** Percentage of adolescents meeting recommendations

Study	Country	Recommendation	% Meeting Recommendations
AlAni et al. (2016) [5]	UAE	FAV ≥ 5 times/d	17.8%
AlAni et al. (2016) [5]	Jordan	FAV ≥ 5 times/d	27.1%
AlAni et al. (2016) [5]	Digibouti	FAV ≥ 5 times/d	40.4%
AlAni et al. (2016) [5]	Lebanon	FAV ≥ 5 times/d	25.4%
AlAni et al. (2016) [5]	Morocco	FAV ≥ 5 times/d	39.5%
AlAni et al. (2016) [5]	Yemen	FAV ≥ 5 times/d	14.3%
AlAni et al. (2016) [5]	Tunisia	FAV ≥ 5 times/d	32.5%
AlAni et al. (2016) [5]	Egypt	FAV ≥ 5 times/d	18.9%
AlAni et al. (2016) [5]	Oman	FAV ≥ 5 times/d	29.0%
Ali et al. (2013) [32]	UAE	Vegetables: M: 3, F: 2.5 cup/d Fruits: M: 2, F: 1.5 cup/d	Vegetables: M:0%, F:6.8% Fruits: M: 4.7%, F: 8.3%
Badr et al. (2017) [47]	Kuwait	3 servings V/d & 2 servings F/d	M: 36%, F:19%
Darfour-Oduro et al. (2018) [7]	Jordan	FAV ≥ 5 servings/d	15.7%
	Algeria	FAV ≥ 5 servings/d	20.6%
Darfour-Oduro et al. (2018) [7]	Djibouti	FAV ≥ 5 servings/d	23%
	Syria	FAV ≥ 5 servings/d	8%
	Libya	FAV ≥ 5 servings/d	5.4%
Darfour-Oduro et al. (2018) [7]	Lebanon	FAV ≥ 5 servings/d	15.8%
Darfour-Oduro et al. (2018) [7]	Morocco	FAV ≥ 5 servings/d	29.5%
Darfour-Oduro et al. (2018) [7]	Yemen	FAV ≥ 5 servings/d	8.7%
Darfour-Oduro et al. (2018) [7]	Sudan	FAV ≥ 5 servings/d	10%
Darfour-Oduro et al. (2018) [7]	Tunisia	FAV ≥ 5 servings/d	19.2%
Darfour-Oduro et al. (2018) [7]	Egypt	FAV ≥ 5 servings/d	14%
Darfour-Oduro et al. (2018) [7]	Mauritania	FAV ≥ 5 servings/d	16.9%
El-Ammari et al. (2020) [6]	Morocco	FAV ≥ 5 times/d	39.4%
Pengpid & Peltzer (2019) [4]	Oman	3 servings V/d 2 servings F/d	Vegetables: M:24.6%, F:19.8% Fruits: M: 54.4%, F: 45.1%

Thirty-nine studies assessed the frequency of intake among fourteen countries. Daily consumption was prevalent among 7.8–66.3% of the students for vegetables and 4.2–53.7% for fruits. Detailed data on the frequency of consumption is supplied in Additional file 5.

### Variation within Arab countries

Findings show a variation in consumption patterns between countries. In most countries, the prevalence of adolescents meeting the recommendations ranged between 10 and 29%. However, even in countries with a higher percentage of adolescents meeting guidelines, namely Djibouti, Oman, Morocco, and Tunisia, less than half of the adolescents met the recommendations for fruits and vegetables consumption [4–6]. On the other hand, adolescents meeting the recommended amount of fruits and vegetables in other countries, namely Libya, Syria, and Yemen, was lower than 10% [7].

One study in Bahrain and Oman and three studies in Saudi Arabia reported the prevalence of “rarely or never consuming fruits and vegetables” [8–12]. Around 38% of adolescents rarely ate vegetables in Bahrain, and about 28% rarely ate fruits [9]. Approximately 14% reported not eating vegetables in Oman, while 8% never ate fruits [8]. Among adolescents in Saudi Arabia, we found different results; in one study, 17 and 27% of participants did not eat vegetables and fruits, respectively. In another study, no consumption was reported among 40% of the participants [10, 11]. In contrast, a third study reported a 4% prevalence of no consumption in the past week [12]. Furthermore, a study in Syria found that half of the adolescents did not consume any green vegetables in the previous week [13].

Comparison between studies remains inaccurate as various studies considered different cutoff points for adequacy. Nevertheless, consumption among Moroccan adolescents is higher than in most countries, as shown by the indicators explained earlier. However, the mean consumption among Moroccan adolescents was also less than five servings per day.

### The proportion of adolescents meeting recommendations

About half of the Moroccan participants had an adequate vegetable intake. In UAE, 78–85% had enough vegetable consumption, while 62–75% had enough fruit consumption [14]. However, in UAE, fruit consumption decreased by 6% between 2005 and 2016, while vegetable consumption decreased slightly [14]. Moreover, higher consumption percentages were reported among females in the UAE data [14]. Around 23 and 18% of Jordanian adolescents met these cutoff points, respectively [15]. In Syria, the percentages were 56 and 46%, respectively, and about 30 and 64% in Sudan [16, 17].

### Fruit consumption vs. vegetable consumption

Daily fruit intake was less frequent than vegetable intake in most countries. Approximately one-fourth of Bahraini adolescents consumed fruits and vegetables daily [9]. Oman had a lower frequency; less than 5% consumed fruits daily, and less than a tenth consumed vegetables daily [8]. In Palestine, the prevalence of daily vegetable consumption was 30–47%, while daily fruit consumption ranged between 12 and 31% [48]. Data from a study conducted in Syria showed a 15% prevalence of fruit consumption [16]. As for Saudi adolescents, studies seem to reveal different findings, where the daily consumption was reported between 10 and 38% for fruit and 20–23% for vegetables, except for one study (54% for vegetables) [10, 29, 31, 36, 37, 49]. Morocco had the highest prevalence of fruit consumption among all countries, with about 28–43% consuming fruits and 49% consuming vegetables daily [30]. In Iraq, the daily consumption of vegetables was 46–62%, and of fruits, 24–46% [46].

On the other hand, adequate fruit intake was more prevalent than vegetables in some countries. For example, in Kuwait, daily consumption of fruit (33–38%) was higher than vegetables (17–22%) [47]. Daily fruits and vegetables consumption was similar among Lebanese adolescents, whose prevalence was around 45 and 44%, respectively [50].

## Discussion

### Low fruits and vegetables consumption

This is, to the best of our knowledge, the first systematic review of fruits and vegetables consumption among adolescents in Arab countries. According to this review, adolescents in Arab countries do not consume the recommended amount of fruits and vegetables. Instead, most countries consumed fruits and vegetables six to eleven times per week, with a daily average of 1–2 times or 1–2 servings. The inadequacy of FAV suggests that Arab countries are at a higher risk of non-communicable diseases, malnutrition, and increased prevalence [31].

Our findings agree with previous research. FAV consumption among adolescents in 49 low-income countries was studied in a comparative study. The majority of Middle Eastern and North African adolescents did not meet FAV recommendations [7]. This is consistent with global findings from the United States and Europe, which found that adolescents’ intakes of fruits and vegetables are significantly lower than recommended levels [51].

Despite the fact that dietary preferences develop throughout adolescence, low FAV consumption may be attributed to environmental factors; low availability of FAV at the household level is a significant potential factor. Despite the scarcity of studies on availability in the Arab region, one global study found that 57% of adults in 18 countries, including Palestine and the UAE, could not afford five servings of fruits and vegetables [52]. Furthermore, in most Arab countries, the availability of FAV in schools is insufficient, if at all. Adolescents’ access to FAV in terms of presentation and preparation is also important. Adolescents consumed more FAV when it was more available and accessible to them by their family or school, according to evidence from non-Arab countries [53].

### Variation between and within Arab countries

Because of their reliance on imports, most Arab countries have a volatile FAV market [54]. Due to water scarcity, reduced agricultural support, limited FAV production, and volatile prices, the availability and affordability of FAV on a national and regional scale have been unstable [55]. Nonetheless, our findings indicate some differences between countries. Better consumption in some countries may be explained by higher agriculture, better socioeconomic status, and lower urbanization. This, however, is insufficient because lower consumption is inconsistent with increased urbanization across countries [56]. Inconsistency was also discovered in terms of national affordability levels [52]. Income appears to have a double-edged effect; on the one hand, countries with higher-income individuals would have better FAV affordability [52]. On the other hand, higher income levels may be associated with a greater prevalence of Western dietary patterns.

Within-country variation in our data adds to the incomparability of countries in this review. Participants’ demographics in the included studies varied greatly. Peer pressure, parent-child relationships, and dependency all have an impact on different age groups. Furthermore, the extracted data included consumption in urban, semi-urban, and rural areas, which may contribute to variation within countries, as previously discovered [57]. Despite the available data, within-country variation makes an evidence-based discussion of Arab region differences difficult.

### Impact of inadequate fruits and vegetables consumption

Low fruits and vegetables intake was a leading global dietary risk factor contributing to one-tenth of disability-adjusted life years (DALYs) in 2010 [58]. A meta-analysis of sixteen prospective cohort studies indicated an association between higher fruits and vegetables consumption and a lower tendency of all-cause mortality and cardiovascular mortality [59]. Another systematic review reported higher blood pressure levels in later life among those who consumed fewer fruits and vegetables [60]. Furthermore, systematic reviews showed a preventive effect of fruits and vegetables intake on early adulthood and postmenopausal breast cancer [61, 62].

Cohort studies focusing on the long-term consequences of a low fruits and vegetables diet across Arab countries are rare. However, around 20% of adolescents were overweight across seven Arab countries [63]. In the previous decade, higher cardiovascular disease-related deaths were observed among Arab adolescents than globally, with higher proportions due to diabetes and cancer [64]. Moreover, cardiovascular diseases (CVDs) were a leading cause of death among older adolescents in middle-income Arab countries [64]. Among other health issues, overweight, obesity, and metabolic conditions are more prevalent in Arab countries compared to global data [64]. A poor diet is a significant risk factor for non-communicable diseases; thus, our findings highlighted inadequate FAV consumption as a public health concern.

### Fruit consumption vs. vegetable consumption

Vegetables’ lower cost and greater affordability may explain why they are consumed more than fruits in some countries [52]. This finding could be applicable to Arab countries. However, food preferences may explain why some countries consume more fruits than vegetables. According to some studies, food preferences strongly predict food choices [65].

### Research on fruits and vegetables consumption among adolescents in Arab countries

The availability of research on fruits and vegetables consumption among adolescents varied by country. For example, we discovered many eligible studies in Saudi Arabia but none in Qatar, Somalia, or Comoros. As a result of our risk of bias assessment, the overall quality of the current evidence is rated as moderate [21]. (See Additional file 4).

Additional data on gender-specific consumption were available in some countries. Three studies conducted in Iraq, the United Arab Emirates, and Saudi Arabia, for example, found that females consumed more fruits and vegetables. In Iraq, the percentage of females reporting a daily intake of fruits and vegetables was around 20% higher. Five studies conducted in Saudi Arabia, Morocco, Kuwait, and Palestine, on the other hand, found that males consumed more FAV than females.

### Limitations

There are several limitations to the current systematic review. First, the tools used to assess fruits and vegetables consumption varied across studies (e.g., some used an FFQ, while others used dietary recall), making a comparison of outcomes difficult. Furthermore, even among papers that used the same tool, the reference period and response categories varied. Second, data heterogeneity is a significant disadvantage because it makes comparing studies difficult. Furthermore, the consumption indicators presented varied across papers (e.g., some studies reported means; others reported percentages, while others reported frequencies). Finally, we noticed unit variation among similar indicators.

The validity and reliability of the instrument were unclear in some studies, affecting data quality. Furthermore, the heterogeneous age ranges constrained the initially planned conclusions of variations across age groups. Our search was limited to databases and excluded data that could be found in grey literature. Furthermore, methodological biases are common, and the representation of the target population at the national level and the generalization of results remain a risk.

Finally, the purpose of this study was to assess FAV consumption among adolescents in Arab countries. More research into the underlying causes of low consumption is needed. A multi-level investigation of the determinants and risk factors is recommended.

## Conclusion

This review highlights an inadequate intake of fruits and vegetables among adolescents in Arab League countries despite the quality of our evidence being rated medium. Our findings underline this critical public health issue. This inadequate consumption may increase the risk of overweight, obesity; metabolic conditions; CVDs, and diabetes. We recommend developing evidence-based programs and policies to improve FAV consumption. Furthermore, we require a better understanding based on valid, representative high-quality data. We also suggest further research investigating the factors behind such a pattern.

## Supplementary Information

**Additional file 1.** PRISMA Checklist.

**Additional file 2.** Overview of included papers [66–72].

**Additional file 3.** Dietary assessment methodologies [66–72].

**Additional file 4.** Risk of bias ratings [66–72].

**Additional file 5.** Frequency of consumption [66–72].

## Data Availability

The final data generated during this study are included in this article and its supplementary information files. In addition, primary datasets are available from the corresponding author upon reasonable request.

## Abbreviations

CVDs: Cardiovascular Diseases
EMR: Eastern Mediterranean Region
FAV: Fruits and Vegetables
FFQ: Food Frequency Questionnaire
GSHS: The Global School-based Student Health Survey
NCDs: Non-Communicable Diseases
PROSPERO: The International Prospective Register of Systematic Reviews
UAE: United Arab Emirates
WHO: World Health Organization
