# Supplementary material for: Consumption of fruits and vegetables among adolescents in Arab Countries: a systematic review
Source: Int J Behav Nutr Phys Act. 2023 Jan 9;20:3. doi: 10.1186/s12966-022-01398-7 (PMC9830827; doi:10.1186/s12966-022-01398-7)
Supplement: Supplementary file 2 — Additional file 2. Overview of included papers [66–72]. [file 12966_2022_1398_MOESM2_ESM.docx]

| Author (year) | Title | Year | Country | outcomes | Design | sample size | Age | Gender |
| --- | --- | --- | --- | --- | --- | --- | --- | --- |
| Abbass et al (2019) ^(66)^ | The prevalence of dental caries among Egyptian children and adolescences and its association with age, socioeconomic status, dietary habits and other risk factors. A cross-sectional study | 2017- 2018 | Egypt | Dietary habits | cross-sectional | 369 | 13 to 18 | M & F |
| Abudayya et. Al (2009) ^(33)^ | Sociodemographic correlates of food habits among school adolescents (12-15 year) in North Gaza Strip | 2002 | Palestine | Meal patterns & food intake | cross-sectional | 944 | 12 to 15 | M & F |
| Abudayya et. Al (2011) ^(34)^ | Diet, nutritional status and school performance among adolescents in Gaza Strip | 2002 | Palestine | Eating behavior, FAV intake | cross-sectional | 932 | 12 to 15 | M & F |
| Abu-Mweis et. al (2014) ^(35)^ | Eating habits, physical activity, and sedentary behaviors of Jordanian adolescents' residents of Amman | 2009 | Jordan | Eating habits | cross-sectional (ATLS) | 735 | 14 to 20 | M & F |
| Aedh et al (2019) ^(29)^ | Iron Deficiency Anemia and Associated Risk Factors among Teenagers in Najran, Saudi ArabiaIron Deficiency Anemia and Associated Risk Factors among Teenagers in Najran, Saudi Arabia | 2018 | Saudi Arabia | Iron deficiency risk factors | cross-sectional | 240 | 13 to 19 | F |
| AlAni et al (2016) ^(5)^ | Consumption of fruits and vegetables among adolescents: a multi-national comparison of eleven countries in the Eastern Mediterranean Region | 2005- 2009 | Oman (11 countries) | % Consuming FAV ≥5 times/d | Cross-Sectional  (GSHS) | 26 328  (11 countries) | 13 to 15 | M & F |
| AlAni et al (2016) ^(5)^ | Consumption of fruits and vegetables among adolescents: a multi-national comparison of eleven countries in the Eastern Mediterranean Region | 2005 - 2009 | UAE  (11 countries) | % Consuming FAV ≥5 times/d | Cross-Sectional  (GSHS) | 26 328  (11 countries) | 13 to 15 | M & F |
| AlAni et al (2016) ^(5)^ | Consumption of fruits and vegetables among adolescents: a multi-national comparison of eleven countries in the Eastern Mediterranean Region | 2005 - 2009 | Jordan (11 countries) | % Consuming FAV ≥5 times/d | Cross-Sectional  (GSHS) | 26 328  (11 countries) | 13 to 15 | M & F |
| AlAni et al (2016) ^(5)^ | Consumption of fruits and vegetables among adolescents: a multi-national  comparison of eleven countries in the Eastern Mediterranean Region | 2005 - 2009 | Digibouti  (11 countries) | % Consuming FAV ≥5 times/d | Cross-Sectional  (GSHS) | 26 328  (11 countries) | 13 to 15 | M & F |
| AlAni et al (2016) ^(5)^ | Consumption of fruits and vegetables among adolescents: a multi-national comparison of eleven countries in the Eastern Mediterranean Region | 2005- 2009 | (Libya) eleven countries  in EMR | % Consuming FAV ≥5 times/d | Cross-Sectional  (GSHS) | 26 328  (11 countries) | 13 to 15 | M & F |
| AlAni et al (2016) ^(5)^ | Consumption of fruits and vegetables among adolescents: a multi-national comparison of eleven countries in the Eastern Mediterranean Region | 2005 - 2009 | Lebanon  (11 countries) | % Consuming FAV ≥5 times/d | Cross-Sectional  (GSHS) | 26 328  (11 countries) | 13 to 15 | M & F |
| AlAni et al (2016) ^(5)^ | Consumption of fruits and vegetables among adolescents: a multi-national comparison of eleven countries in the Eastern Mediterranean Region | 2005- 2009 | Morocco | % Consuming FAV ≥5 times/d | Cross-Sectional  (GSHS) | 26 328  (11 countries) | 13 to 15 | M & F |
| AlAni et al (2016) ^(5)^ | Consumption of fruits and vegetables among adolescents: a multi-national comparison of eleven countries in the Eastern Mediterranean Region | 2005 - 2009 | Yemen (11 countries) | % Consuming FAV ≥5 times/d | Cross-Sectional  (GSHS) | 26 328  (11 countries) | 13 to 15 | M & F |
| AlAni et al (2016) ^(5)^ | Consumption of fruits and vegetables among adolescents: a multi-national comparison of eleven countries in the Eastern Mediterranean Region | 2005 - 2009 | Tunisia  (11 countries) | % Consuming FAV ≥5 times/d | Cross-Sectional  (GSHS) | 26 328  (11 countries) | 13 to 15 | M & F |
| AlAni et al (2016) ^(5)^ | Consumption of fruits and vegetables among adolescents: a multi-national comparison of eleven countries in the Eastern Mediterranean Region | 2005 - 2009 | Egypt (11countries) | % Consuming FAV ≥5 times/d | Cross-Sectional  (GSHS) | 26 328  (11 countries) | 13 to 15 | M & F |
| AlBuhairan et al (2015) ^(67)^ | Time for an Adolescent Health Surveillance System in Saudi Arabia: Findings from "jeeluna" | Not mentioned | Saudi Arabia | Health risk behaviors | cross-sectional | 12,575 | Mean: 15.8 | M & F |
| Alghadir et al (2016) ^(12)^ | Television watching, diet and body mass index of school children in Saudi Arabia | Not mentioned | Saudi Arabia | Eating habits and diet preferences | Not mentioned (cross-sectional) | 214 | 12 to 16 | M & F |
| Al-Hazzaa et al (2011) ^(36)^ | Physical activity, sedentary behaviors and dietary habits among Saudi adolescents relative to age, gender and region | 2009 | Saudi Arabia | Dietary habits | cross-sectional (ATLS) | 2908 | 14 to 19 | M & F |
| Al-Hazzaa et al (2013) ^(37)^ | A cross-cultural comparison of health behaviors between Saudi and British adolescents living in urban areas: gender by country analyses | 2009/2010 | Saudi Arabia  & Britain | Health behaviors | cross-sectional | 1,648  (Saudi Arabia) | 14 to 18 | M & F |
| Aljuaid et al (2020) ^(51)^ | The prevalence of obesity among school students and its relation to dietary and physical habits | 2014- 2015 | Saudi Arabia | Dietary habits | cross-sectional | 2943 | Mean: 15.4 | Mostly M |
| Ali et al (2013) ^(32)^ | High proportion of 6 to 18-year-old children and adolescents in the United Arab Emirates are not meeting dietary recommendations | Not mentioned | UAE | Adequacy, and Diet quality | cross-sectional | 529 | 6 to 18 | M & F |
| Allafi et al (2014) ^(38)^ | Physical activity, sedentary behaviours and dietary habits among Kuwaiti adolescents: gender differences | 2009 | Kuwait | Dietary habits | cross-sectional | 906 | 14 to 19 | M & F |
| AlSabbah et al (2007) ^(50)^ | Food habits and physical activity patterns among Palestinian adolescents: findings from the national study of Palestinian schoolchildren  (HBSC-WBG2004) | 2004 | Palestine | Food habits | cross-sectional  (HBSC) | 8885 | 12 to 18 | M & F |
| Al-Sagarat et al (2017) ^(39)^ | Prevalence of Health-risk Behaviours among Government Schools' Students in Jordan | 2016 | Jordan | Health risk behaviors | cross-sectional | 1256 | 13 to 16 | M & F |
| Al-Sheyab et al (2018) ^(68)^ | Unhealthy eating habits among adolescent waterpipe smokers in Jordan: The Irbid-TRY study | Not mentioned | Jordan | Eating habits | cross-sectional  (Irbid-TRY study) | 1771 | Mean: 14.6 | M & F |
| Alsubaie et al (2018) ^(51)^ | Intake of fruit, vegetables and milk products and correlates among school boys in Saudi Arabia | 2013 | Saudi Arabia | FAV consumption | cross-sectional | 725 | 7 to12 | M |
| Alzahrani et. Al 2017 ^(69)^ | Psychosocial determinants of clustering health-compromising behaviors among Saudi male adolescents | Not Mentioned | Saudi Arabia | Fruit consumption | cross-sectional | 1335 | 13- 14  & 17-19 | Males |
| Alzahrani et al (2014) ^(31)^ | Patterns of clustering of six health-compromising behaviours in Saudi adolescents | Not mentioned | Saudi Arabia | Fruit consumption | Not mentioned (cross-sectional) | 1,335 | 13 to 14  & 17 to 19 | M |
| Amahmid et al (2019) ^(30)^ | Nutrition education in school curriculum: impact on adolescents’ attitudes and dietary behaviours | Not mentioned | Morocco | Nutrition behaviors | Not mentioned (cross-sectional) | 120 | 13 and 15 | M & F |
| Anwar et al. (2018) ^(8)^ | Were the numerator(s) and denominator r(s) for the parameter of interest appropriate? | Not mentioned | Oman | Eating habits | cross-sectional | 359 | Not mentioned | F |
| Aounallah-Skhiri et al  (2011) ^(40)^ | Nutrition transition among adolescents of a south-Mediterranean country: dietary patterns, association with socio-economic factors, overweight and  blood pressure. A cross-sectional study in Tunisia | 2005 | Tunisia | Dietary intake | cross sectional | 1019 | 15 to 19 | M & F |
| Azekour et al (2019) ^(41)^ | Socioeconomic characteristics and fruit/vegetable intakes among scholar children in the oasis of Tafilalet, Southeastern Morocco | 2015- 2017 | Morocco | FAV consumption | cross-sectional | 3 684 | 5 to 9  10 to12  >= 13 | M & F |
| Badr et al (2017) ^(47)^ | Differences in physical activity, eating habits and risk of obesity among Kuwaiti adolescent boys and girls: a population-based study | 2011 | Kuwait | Food choices | cross-sectional | 2672 | 13 to 15 | M & F |
| Bashour et. Al (2004) ^(13)^ | Survey of dietary habits of in-school adolescents in Damascus, Syrian Arab Republic | 2001 | Syria | Dietary habits | cross-sectional | 3507 | 13 to 18 | M & F |
| Chacar et al (2011) ^(42)^ | Public schools adolescents' obesity and growth curves in Lebanon | 2007 | Lebanon | Food consumption | cross-sectional | 2547 | 11 to 18 | M & F |
| Collison et al (2010) ^(43)^ | Sugar-sweetened carbonated beverage consumption correlates with BMI, waist circumference, and poor dietary choices in school children | 2007 | Saudi Arabia | Dietary habits | cross-sectional | 9433 | 10 to 19 | M & F |
| Darfour-Oduro et al (2018) ^(48)^ | A comparative study of fruit and vegetable consumption and physical activity among adolescents in 49 Low-and-Middle-Income Countries | 2004- 2013. | Jordan (49 LMICs) | FAV consumption patterns | Cross-Sectional  (GSHS) | 164,771  (total) | 13 to 15 | M & F |
| Darfour-Oduro et al (2018) ^(48)^ | A comparative study of fruit and vegetable consumption and physical activity among adolescents in 49 Low-and-Middle-Income Countries | 2004 -2013 | Algeria  (49 LMICs) | FAV consumption patterns | Cross-Sectional  (GSHS) | 164,771  (total) | 13 to 15 | M & F |
| Darfour-Oduro et al (2018) ^(48)^ | A comparative study of fruit and vegetable consumption and physical activity among adolescents in 49 Low-and-Middle-Income Countries | 2004 - 2013 | Digibouti  (49 LMICs0 | FAV consumption patterns | Cross-Sectional  (GSHS) | 164,771  (total) | 13 to 15 | M & F |
| Darfour-Oduro et al (2018) ^(48)^ | A comparative study of fruit and vegetable consumption and physical activity among adolescents in 49 Low-and-Middle-Income Countries | 2004 - 2013 | Syria  (49 LMICs) | FAV consumption patterns | Cross-Sectional  (GSHS)) | 164,771  (total) | 13 to 15 | M & F |
| Darfour-Oduro et al (2018) ^(48)^ | A comparative study of fruit and vegetable consumption and physical activity among adolescents in 49 Low-and-Middle-Income Countries | 2004 - 2013. | (Libya) 49 LMICs | FAV consumption | Cross-Sectional  (GSHS) | 164,771  (total) | 13 to 15 | M & F |
| Darfour-Oduro et al (2018) ^(48)^ | A comparative study of fruit and vegetable consumption and physical activity among adolescents in 49 Low-and-Middle-Income Countries | 2004 - 2013 | (Lebanon) 49 LMICs | FAV consumption | Cross-Sectional  (GSHS) | 164,771  (total) | 13 to 15 | M & F |
| Darfour-Oduro et al (2018) ^(48)^ | A comparative study of fruit and vegetable consumption and physical activity among adolescents in 49 Low-and-Middle-Income Countries | 2004- 2013 | (Morocco) in 49 LMICs | FAV consumption | Cross-Sectional  (GSHS) | 164,771  (total) | 13 to 15 | M & F |
| Darfour-Oduro et al (2018) ^(48)^ | A comparative study of fruit and vegetable consumption and physical activity among adolescents in 49 Low-and-Middle-Income Countries | 2004 - 2013 | Yemen (49 LMICs) | FAV consumption | Cross-Sectional  (GSHS) | 164,771  (total) | 13 to 15 | M & F |
| Darfour-Oduro et al (2018) ^(48)^ | A comparative study of fruit and vegetable consumption and physical activity among adolescents in 49 Low-and-Middle-Income Countries | 2004 - 2013. | (Sudan) 49 LMICs | FAV consumption | Cross-Sectional  (GSHS) | 164,771  (total) | 13 to 15 | M & F |
| Darfour-Oduro et al (2018) ^(48)^ | A comparative study of fruit and vegetable consumption and physical activity among adolescents in 49 Low-and-Middle-Income Countries | 2004 - 2013 | Tunisia  (49 LMICs) | FAV consumption | Cross-Sectional  (GSHS) | 164,771  (total) | 13 to 15 | M & F |
| Darfour-Oduro et al (2018) ^(48)^ | A comparative study of fruit and vegetable consumption and physical activity among adolescents in 49 Low-and-Middle-Income Countries | 2004 - 2013 | (Egypt) in 49 LMICs | FAV consumption | Cross-Sectional  (GSHS) | 164,771  (total) | 13 to 15 | M & F |
| Darfour-Oduro et al (2018) ^(48)^ | A comparative study of fruit and vegetable consumption and physical activity among adolescents in 49 Low-and-Middle-Income Countries | 2004 - 2013 | (Moritanya) in 49 LMICs | FAV consumption | Cross-Sectional  (GSHS) | 164,771  (total) | 13 to 15 | M & F |
| ElAchhab et al (2018) ^(70)^ | Physical inactivity, sedentary behaviors and dietary habits among Moroccan adolescents in secondary school | 2014 | Morocco | Dietary habits | cross-sectional | 346 | 14 to 19 | M & F |
| El-Kassas et al (2017) ^(52)^ | The Dual Burden of Malnutrition and Associated Dietary and Lifestyle Habits among Lebanese School Age Children Living in Orphanages in North Lebanon | 2015 | North Lebanon | Nutritional status | cross-sectional | 153 | 5 to 14  (10-14) | M & F |
| El-Ammari et al (2020) ^(6)^ | Social-ecological influences on unhealthy dietary behaviors among Moroccan adolescents: a mixed-methods study | 2016 | Morocco | Dietary behaviors Dietary behaviors | cross-sectional | 764 | 14 to 19 | M & F |
| Gharib et. al (2011) ^(71)^ | Energy and macronutrient intake and dietary pattern among school children in Bahrain: a cross-sectional study | 1999-2001 | Bahrain | Food frequency | cross-sectional | 2594 | 6 to 18 | M & F |
| Ghrayeb et al (2014) ^(44)^ | Non-communicable diseases behavioral risk factors among Palestinian adolescents: A descriptive study from a rural community of Tarqumia | 2011 | Palestine | Dietary behaviors | cross-sectional | 720 | 13 to 17 | M & F |
| Haddad et (2009) ^(15)^ | Wellness appraisal among adolescents in Jordan: a model from a developing country: a cross-sectional questionnaire survey | Not mentioned | Jordan | Nutrition & habits | cross sectional | 530 | 12 to 17 | M & F |
| Hamrani et al (2015) ^(72)^ | Physical activity and dietary habits among Moroccan adolescents | 2011 | Morocco | Dietary habits | cross-sectional | 669 | 15 to 19.9 | M & F |
| Mikki et al (2010) ^(45)^ | Dietary habits of Palestinian adolescents and associated sociodemographic characteristics in Ramallah, Nablus and Hebron governorates | 2005 | Palestine | Food habits | Cross-sectional | 2952 | 13 to 15 | M & F |
| Musaiger et al (2011) ^(9)^ | Dietary and lifestyle habits amongst adolescents in Bahrain | 2006 | Bahrain | Dietary habits | cross-sectional | 735 | 15 to 18 | M & F |
| Musaiger et al (2014) ^(16)^ | Dietary habits and lifestyle among adolescents in Damascus, Syria | 2012 | Syria | Dietary habits | cross sectional | 365 | 15 to 18 | M & F |
| Musaiger et al (2014) ^(46)^ | Eating habits, inactivity, and sedentary behavior among adolescents in Iraq: sex differences in the hidden risks of noncommunicable diseases | 2009 | Iraq | Eating habits | Cross-sectional  (ATLS) | 723 | 15 to 18 | M & F |
| Musaiger et al (2016) ^(17)^ | Obesity, Dietary Habits, and Sedentary Behaviors Among Adolescents in Sudan: Alarming Risk Factors for Chronic Diseases in a Poor Country | 2013 - 2014 | Sudan | Dietary habits | cross-sectional | 945 | 14 to 18 | M & F |
| Mahfouz te al (2011) ^(11)^ | Nutrition, physical activity, and gender risks for adolescent obesity in Southwestern Saudi Arabia | 2008 | Saudi Arabia | Obesity related behavior | cross-sectional | 1,869 | 11 to 19 | M & F |
| Mahfouz et al (2012) ^(10)^ | Gender differences in cardiovascular risk factors among adolescents in Aseer Region, southwestern Saudi Arabia | Not mentioned | Saudi Arabia | Cardiovascular risk factors | cross-sectional | 1869 | 12 to 19 | M & F |
| Pengpid & Peltzer (2019) ^(4)^ | Trends of dietary behavior, physical activity, interpersonal violence and hand hygiene behavior among school-going adolescents in Oman: cross-sectional national surveys from 2005, 2010 and 2015 | 2005 | Oman | Dietary behavior | Cross-sectional  (GSHS) | 8053 | Mean: 14.8 | M & F |
| Pengpid et al (2020) ^(14)^ | Trends in the prevalence of twenty health indicators among adolescents in United Arab Emirates: cross-sectional national school surveys from 2005, 2010 and 2016 | 2005, 2010 & 2016 | UAE | Health and protective indicators | cross-sectional | 24,220 | Median:14 | M & F |
