# Supplementary material for: Consumption of fruits and vegetables among adolescents in Arab Countries: a systematic review
Source: Int J Behav Nutr Phys Act. 2023 Jan 9;20:3. doi: 10.1186/s12966-022-01398-7 (PMC9830827; doi:10.1186/s12966-022-01398-7)
Supplement: Supplementary file 3 — Additional file 3. Dietary assessment methodologies [66–72]. [file 12966_2022_1398_MOESM3_ESM.docx]

| Study | Dietary Assessment Methodology | Dietary Measurement | Dietary Variables | Validity of the Dietary Measure | Reliability of the Dietary Measure |
| --- | --- | --- | --- | --- | --- |
| Abbass et al (2019) ^(66)^ | questionnaire | questions on dietary habits | fruits, vegetables, and other foods | Not mentioned | Not mentioned |
| Abudayya et. Al (2009) ^(33)^ | self-administered questionnaire | Food frequency questionnaire (FFQ) | 42 food items including fruits and vegetables | Not validated | Not mentioned |
| Abudayya et. Al (2011) ^(34)^ | self- administered questionnaire | Food frequency questionnaire (FFQ) | 42 food items including fruits and vegetables | Not validated | Not mentioned |
| Abu-Mweis et. al (2014) ^(35)^ | self-administered questionnaire | Not  Mentioned | fruits, vegetables  and other foods | Previously validated | Not  Mentioned |
| Aedh et al (2019) ^(29)^ | a pretested questionnaire | Not mentioned | fruits, vegetables, and other foods | Not mentioned | Not mentioned |
| AlAni et al (2016) ^(5)^ | a self-administered questionnaire | questions on dietary behaviors | Fruits and Vegetables | Not mentioned (only translation) pilot tested | Not mentioned |
| AlAni et al (2016) ^(5)^ | a self-administered questionnaire | questions on dietary behaviors | Fruits and Vegetables | Not mentioned (only translation) pilot tested | Not mentioned |
| AlAni et al (2016) ^(5)^ | a self-administered questionnaire | questions on dietary behaviors | Fruits and Vegetables | Not mentioned (only translation) pilot tested | Not mentioned |
| AlAni et al (2016) ^(5)^ | a self-administered questionnaire | questions on dietary behaviors | Fruits and Vegetables | Not mentioned (only translation) pilot tested | Not mentioned |
| AlAni et al (2016) ^(5)^ | a self-administered questionnaire | questions on dietary behaviors | Fruits and Vegetables | Not mentioned (only translation) pilot tested | Not mentioned |
| AlAni et al (2016) ^(5)^ | a self-administered questionnaire | questions on dietary behaviors | Fruits and Vegetables | Not mentioned (only translation) pilot tested | Not mentioned |
| AlAni et al (2016) ^(5)^ | a self-administered questionnaire | questions on dietary behaviors | Fruits and Vegetables | Not mentioned (only translation) pilot tested | Not mentioned |
| AlAni et al (2016) ^(5)^ | a self-administered questionnaire | questions on dietary behaviors | Fruits and Vegetables | Not mentioned (only translation) pilot tested | Not mentioned |
| AlAni et al (2016) ^(5)^ | a self-administered questionnaire | questions on dietary behaviors | Fruits and Vegetables | Not mentioned (only translation) pilot tested | Not mentioned |
| AlAni et al (2016) ^(5)^ | a self-administered questionnaire | questions on dietary behaviors | Fruits and Vegetables | Not mentioned (only translation) pilot tested | Not mentioned |
| AlBuhairan et al (2015) ^(67)^ | self-administered questionnaire | including questions on  nutrition/dietary behaviors; | fruits and vegetables | Not mentioned used the GSHS questionnaire, validated | Not mentioned |
| Alghadir et al (2016) ^(12)^ | self-administered  online questionnaire | questions on daily diet pattern | fruits , and other foods | Not mentioned | Not mentioned |
| Al-Hazzaa et al (2011) ^(36)^ | questionnaire | ten specific questions: the frequency of certain dietary habits | vegetables (cooked and uncooked), and fruits and other foods | Not mentioned | Reliable |
| Al-Hazzaa et al (2013) ^(37)^ | self-reported questionnaire | questions on dietary habits | fruits , vegetables  and other foods | Validated  Acceptable validity | high reliability |
| Ali et al (2013) ^(32)^ | interviews | single 24-hour food recall | vegetables, fruit juices, and other foods | standardized process was used | Not mentioned |
| Aljuaid et al (2020) ^(51)^ | questionnaire | questions on dietary habits | fruits, vegetables, and other foods healthy and unhealthy foods | Validated | Not mentioned |
| Allafi et al (2014) ^(38)^ | the ALTS questionnaire | consisting of ten questions was used to assess certain dietary habits (healthy and unhealthy) | vegetables (cooked and uncooked), fruit, and other foods | Validated | Not mentioned |
| AlSabbah et al (2007) ^(50)^ | A self-report questionnaire | questions on food consumption | fruits , vegetables, and other foods | The questionnaire was translated into the native language (Arabic) and piloted on 300 students | Not mentioned |
| Al-Sagarat et al (2017) ^(39)^ | self-reported questionnaire  The modified Arabic version of the GSHS | Eating habits (12 questions) | fruits , vegetables  and other foods | The validity of the Arabic version of the GSHS were assessed by numerous studies conducted in different Arabic countries including Jordan | Reliable |
| Al-Sheyab et al (2018) ^(68)^ | self-reported  the Students As Lifestyle Activists survey (SALSA) | items concerning eating habits | fruits, vegetables, and other foods | Validity analyses was done in an Arabic-speaking population (data are not yet published) | Reliable |
| Alsubaie et al (2018) ^(51)^ | a self-reported questionnaire | questions on dietary consumption | fruits , vegetables and dairy | GSHS questionnaire, validated  also, also validated among schoolchildren of a similar age to the study participants, prior to the main study throughout a pilot study | Not mentioned |
| Alzahrani et al (2014) ^(31)^ | self-administered classroom-based questionnaire | questions on dietary behaviors | fruit and sweets | Not mentioned  Adapted from a previously used questionnaire, translated, and piloted | Not mentioned |
| Alzahrani et. Al 2017 ^(69)^ | self- administered questionnaire | two items on dietary behaviors  questionnaire | fruit & sweets | Not mentioned Translation, reviewed, and piloted | Not  Mentioned |
| Amahmid et al (2019) ^(30)^ | questionnaire | questions on dietary habits | fruits , vegetables  and other foods | Not mentioned | Not mentioned |
| Anwar et al. (2018) ^(8)^ | A pretested, structured questionnaire | questions on food eating habits | fruits, vegetables, and others | Validated after piloting it in one school at the start of the study | Not mentioned |
| Aounallah-Skhiri et al  (2011) ^(40)^ | self-reported  questionnaire | semi-quantitative food frequency questionnaire (FFQ) | The 134 food items of the food frequency questionnaire grouped into 43 food groups | Validated | Not mentioned |
| Azekour et al (2019) ^(41)^ | questionnaire | a 24-hour recall | all foods consumed the previous day | Not mentioned | Not mentioned |
| Badr et al (2017) ^(47)^ | The Kuwait GSHS  questionnaire | food choices and eating habits  in the last 30 days preceding | fruits , vegetables  and other foods | Not mentioned, (only translation) | Not mentioned |
| Bashour et. Al (2004) ^(13)^ | self-administered questionnaire | frequency of the main food items consumed in the past week | main food items including fruits and vegetables | Research has been done to improve the validity | research has been done to improve the reliability |
| Chacar et al (2011) ^(42)^ | self- completed questionnaire | food frequency questionnaire | Salads, fruits, and other foods | Not mentioned | Not mentioned |
| Collison et al (2010) ^(43)^ | Questionnaire self-reported | Food Frequency Questionnaire(FFQ) reference period: 7 days | 21 food items  including fruits and vegetables | Not mentioned | Not mentioned |
| Darfour-Oduro et al (2018) ^(48)^ | GSHS questionnaire | following GSHS questions | fruits and vegetables | GSHS questionnaire is validated | Not mentioned |
| Darfour-Oduro et al (2018) ^(48)^ | GSHS questionnaire | following GSHS questions | fruits and vegetables | GSHS questionnaire is validated | Not mentioned |
| Darfour-Oduro et al (2018) ^(48)^ | GSHS questionnaire | following GSHS questions | fruits and vegetables | GSHS questionnaire is validated | Not mentioned |
| Darfour-Oduro et al (2018) ^(48)^ | GSHS questionnaire | following GSHS questions | fruits and vegetables | GSHS questionnaire is validated | Not mentioned |
| Darfour-Oduro et al (2018) ^(48)^ | GSHS questionnaire | following GSHS questions | fruits and vegetables | GSHS questionnaire is validated | Not mentioned |
| Darfour-Oduro et al (2018) ^(48)^ | GSHS questionnaire | following GSHS questions | fruits and vegetables | GSHS questionnaire is validated | Not mentioned |
| Darfour-Oduro et al (2018) ^(48)^ | GSHS questionnaire | following GSHS questions | fruits and vegetables | GSHS questionnaire is validated | Not mentioned |
| Darfour-Oduro et al (2018) ^(48)^ | GSHS questionnaire | following GSHS questions | fruits and vegetables | GSHS questionnaire is validated | Not mentioned |
| Darfour-Oduro et al (2018) ^(48)^ | GSHS questionnaire | following GSHS questions | fruits and vegetables | GSHS questionnaire is validated | Not mentioned |
| Darfour-Oduro et al (2018) ^(48)^ | GSHS questionnaire | following GSHS questions | fruits and vegetables | GSHS questionnaire is validated | Not mentioned |
| Darfour-Oduro et al (2018) ^(48)^ | GSHS questionnaire | following GSHS questions | fruits and vegetables | GSHS questionnaire is validated | Not mentioned |
| Darfour-Oduro et al (2018) ^(48)^ | GSHS questionnaire | following GSHS questions | fruits and vegetables | GSHS questionnaire is validated | Not mentioned |
| ElAchhab et al (2018) ^(70)^ | The ATLS questionnaire | questions on healthy dietary habits | fruits and vegetables  and other foods | validated | Not mentioned |
| El-Ammari et al (2020) ^(6)^ | for the quantitative data:  e the Moroccan version of the GSHS. | questions on fav consumption  and other dietary behaviors | fruits and vegetables , fast drinks, fast foods | Not mentioned  GSHS questionnaire is validated | Not mentioned |
| El-Kassas et al (2017) ^(52)^ | structured interview questionnaire | a semiquantitative food frequency questionnaire | fruits , vegetables, and other foods | Not mentioned | Not mentioned |
| Gharib et. al (2011) ^(71)^ | questionnaire | a 24-hour dietary recall, by a subsample  and food frequency questionnaire by all | 7 groups of food items  including fruits and vegetables | Quality Controls used | Quality Controls used |
| Ghrayeb et al (2014) ^(44)^ | self- administered questionnaire Arabic version of the international  Global School-Based Health Survey  (GSHS)) | questions on consumption of  fruits and vegetables, soft drinks  and fast foods | fruits and vegetables, soft drinks  and fast foods | Not mentioned   GSHS uestionnaire is validated | Not mentioned |
| Haddad et (2009) ^(15)^ | a questionnaire that includes  7 subscales | eating habits subscale,  10 items related to eating habits | fruits , vegetables  and other foods | The Arabic version of the instrument was pilot tested on a group of Jordanian adolescent students  Content validity of the modifying instrument was assessed by two faculty members both of whom are experts in the area of adolescent’s and children’s health. | Reliable |
| Hamrani et al (2015) ^(72)^ | self-reported  questionnaire | items on two food groups ( healthy and unhealthy) | fruits , vegetables  and other foods (healthy and unhealthy) | Validated | Not mentioned |
| Mahfouz et al (2012) ^(10)^ | The structured questionnaire used in the study was the validated Arabic version of CDC Adolescent Health Survey | the questionnaire included questions on behaviors related food choices | Fresh vegetables fresh fruits fresh juices and other foods | Validated  The questionnaire used was the validated among adolescents in another governorate | Not mentioned |
| Mahfouz te al (2011) ^(11)^ | a structured questionnaire Arabic version of CDC Adolescent  Health Survey | questions on food choices | Fresh vegetables fresh fruits fresh juices and other foods | Validated  The questionnaire used was the validated among adolescents in another governorate | Not mentioned |
| Mikki et al (2010) ^(45)^ | Self-administered student  and parent questionnaires | food frequency lists in the students  questionnaire | fruits, vegetables and other foods,  list of forty-two food items | Not mentioned  piloted with forty girls and twenty-two boys and their parents in two classes (8th and 9th grades) | Reliable |
| Musaiger et al (2011) ^(9)^ | structured self-administered questionnaire | questions on dietary habits | fruits, vegetables  and other foods | Modified from a previously validated questionnaire  Content validity tested | test-retest reliability Pearson correlation: 0.87 |
| Musaiger et al (2014) ^(16)^ | questionnaire | two sections on food frequency intake; dietary habits; | fruits , vegetables  and other foods | Previously pretested validated | Not mentioned |
| Musaiger et al (2014) ^(46)^ | questionnaire | questions on dietary habits | fruits , vegetables, and other foods | Pretested and validated | Acceptable reliability |
| Musaiger et al (2016) ^(17)^ | self-reported pretested questionnaire | questions on intake | fruits, vegetables, and other foods healthy and unhealthy foods | Valid  The validity of the questionnaire was reviewed by 2 nutritional specialists, and then the content validity of the tool was tested by 5 experts in the field of nutrition and public health. | Reliable |
| Pengpid & Peltzer (2019) ^(4)^ | questionnaire | 2 Questions | Fruit & Vegetables | Not mentioned  GSHS questionnaire is validated | 77% agreement Cohen’s kappa coefficient was 0.47 |
| Pengpid et al (2020) ^(14)^ | The GSHS questionnaire | questions on dietary behaviors | Fruits and Vegetables | Not mentioned  GSHS questionnaire is validated | Not mentioned |
