# Supplementary material for: Consumption of fruits and vegetables among adolescents in Arab Countries: a systematic review
Source: Int J Behav Nutr Phys Act. 2023 Jan 9;20:3. doi: 10.1186/s12966-022-01398-7 (PMC9830827; doi:10.1186/s12966-022-01398-7)
Supplement: Supplementary file 4 — Additional file 4. Risk of bias ratings [66–72]. [file 12966_2022_1398_MOESM4_ESM.docx]

| Study | Target population | Sampling frame | Random selection | Response Bias | Direct collection | Case definition | Reliability & Validity | Standard assessment | Prevalence period | Appropriate fraction |
| --- | --- | --- | --- | --- | --- | --- | --- | --- | --- | --- |
| Abbass et al (2019) ^(66)^ |  |  |  |  |  |  |  |  |  |  |
| Abudayya et. Al (2009) ^(33)^ |  |  |  |  |  |  |  |  |  |  |
| Abudayya et. Al (2011) ^(34)^ |  |  |  |  |  |  |  |  |  |  |
| Abu-Mweis et. al (2014) ^(35)^ |  |  |  |  |  |  |  |  |  |  |
| Aedh et al (2019) ^(29)^ |  |  |  |  |  |  |  |  |  |  |
| AlAni et al (2016) ^(5)^ |  |  |  |  |  |  |  |  |  |  |
| AlBuhairan et al (2015) ^(67)^ |  |  |  |  |  |  |  |  |  |  |
| Alghadir et al (2016) (12) |  |  |  |  |  |  |  |  |  |  |
| Al-Hazzaa et al (2011) ^(36)^ |  |  |  |  |  |  |  |  |  |  |
| Al-Hazzaa et al (2013) ^(37)^ |  |  |  |  |  |  |  |  |  |  |
| Ali et al (2013) ^(32)^ |  |  |  |  |  |  |  |  |  |  |
| Aljuaid et al (2020) ^(51)^ |  |  |  |  |  |  |  |  |  |  |
| Allafi et al (2014) ^(38)^ |  |  |  |  |  |  |  |  |  |  |
| AlSabbah et al (2007) ^(50)^ |  |  |  |  |  |  |  |  |  |  |
| Al-Sagarat et al (2017) ^(39)^ |  |  |  |  |  |  |  |  |  |  |
| Al-Sheyab et al (2018) ^(68)^ |  |  |  |  |  |  |  |  |  |  |
| Alsubaie et al (2018) ^(51)^ |  |  |  |  |  |  |  |  |  |  |
| Alzahrani et al (2014) ^(31)^ |  |  |  |  |  |  |  |  |  |  |
| Alzahrani et. Al 2017 ^(69)^ |  |  |  |  |  |  |  |  |  |  |
| Amahmid et al (2019) ^(30)^ |  |  |  |  |  |  |  |  |  |  |
| Anwar et al. (2018) ^(8)^ |  |  |  |  |  |  |  |  |  |  |
| Aounallah-Skhiri et al  (2011) ^(40)^ |  |  |  |  |  |  |  |  |  |  |
| Azekour et al (2019) ^(41)^ |  |  |  |  |  |  |  |  |  |  |
| Badr et al (2017) ^(47)^ |  |  |  |  |  |  |  |  |  |  |
| Bashour et. Al (2004) ^(13)^ |  |  |  |  |  |  |  |  |  |  |
| Chacar et al (2011) ^(42)^ |  |  |  |  |  |  |  |  |  |  |
| Collison et al (2010) ^(43)^ |  |  |  |  |  |  |  |  |  |  |
| Darfour-Oduro et al (2018) ^(48)^ |  |  |  |  |  |  |  |  |  |  |
| ElAchhab et al (2018) ^(70)^ |  |  |  |  |  |  |  |  |  |  |
| El-Ammari et al (2020) ^(6)^ |  |  |  |  |  |  |  |  |  |  |
| El-Kassas et al (2017) ^(52)^ |  |  |  |  |  |  |  |  |  |  |
| Gharib et. al (2011) ^(71)^ |  |  |  |  |  |  |  |  |  |  |
| Ghrayeb et al (2014) ^(44)^ |  |  |  |  |  |  |  |  |  |  |
| Haddad et (2009) ^(15)^ |  |  |  |  |  |  |  |  |  |  |
| Hamrani et al (2015) ^(72)^ |  |  |  |  |  |  |  |  |  |  |
| Mahfouz et al (2012) ^(10)^ |  |  |  |  |  |  |  |  |  |  |
| Mahfouz te al (2011) ^(11)^ |  |  |  |  |  |  |  |  |  |  |
| Mikki et al (2010) ^(45)^ |  |  |  |  |  |  |  |  |  |  |
| Musaiger et al (2011) ^(9)^ |  |  |  |  |  |  |  |  |  |  |
| Musaiger et al (2014) ^(16)^ |  |  |  |  |  |  |  |  |  |  |
| Musaiger et al (2014) ^(46)^ |  |  |  |  |  |  |  |  |  |  |
| Musaiger et al (2016) ^(17)^ |  |  |  |  |  |  |  |  |  |  |
| Pengpid & Peltzer (2019) ^(4)^ |  |  |  |  |  |  |  |  |  |  |
| Pengpid et al (2020) ^(14)^ |  |  |  |  |  |  |  |  |  |  |
