# Supplementary material for: Consumption of fruits and vegetables among adolescents in Arab Countries: a systematic review
Source: Int J Behav Nutr Phys Act. 2023 Jan 9;20:3. doi: 10.1186/s12966-022-01398-7 (PMC9830827; doi:10.1186/s12966-022-01398-7)
Supplement: Supplementary file 5 — Additional file 5. Frequency of consumption [66–72]. [file 12966_2022_1398_MOESM5_ESM.docx]

| Study | Country | Frequency of consumption |  | % |
| --- | --- | --- | --- | --- |
| Gharib et. al (2011) ^(71)^ | Bahrain |  |  | Fruits and vegetables daily:  0.25 |
| Musaiger et al (2011) ^(9)^ | Bahrain | - Vegetables/salad -Daily:  Total: 26.3% M: 52.8% F: 47.2% (p-value 0.125) -1 to 3 times:  Total:26.1% M: 46.4% F: 53.6% -4 to 6 times:  Total: 9.5% M: 44.3% F: 55.7% -rarely:  Total: 38.1% M: 41.8% F: 58.2% | -Fruits -Daily:  Total: 25.3% M: 51.1% F: 48.9% (p-value=0.000) -1 to 3 times:  Total: 32.4% M: 48.3% F: 51.7% -4 to 6 times: Total: 14.6% M: 56.1% F: 43.9% -rarely:  Total: 27.7% M: 33.5% F: 66.5% (p-value=0.001) |  |
| Anwar et al. (2018) ^(8)^ | Oman | Vegetables Never: 13.9% <1 daily: 78.3  >=1 once daily: 7.8% | Fruits Never: 8.4% <1 daily: 87.4%   >=1 once daily: 4.2% |  |
| Pengpid et al (2020) ^(14)^ | UAE | Males: Vegetable < 3 day: 2005: 81.0% 2010: 79.9% 2016: 78.4%   females: -Vegetable <3 day:  2005: 84.7% 2010: 84.8% 2016: 79.5% | Males:  Fruits < 2 day: 2005: 68.7% 2010: 70.6% 2016:61.6%  Fruits <2 day:  2005: 75.2% |  |
| Haddad et (2009) ^(15)^ | Jordan | vegetables  4> 81.7% 4=<18.3% vegetables: time/ day 1 = 51.7% 2-3 = 30.0% 4=< 18.3%l | Fruits 4> 77.5% 4=< 22.5% Fruits: time/day 1 : 44.3% 2-3 : 33.2% 4=<: 22.5% |  |
| Al-Sagarat et al (2017) ^(39)^ | Jordan | - |  | - |
| Al-Sheyab et al (2018) ^(68)^ | Northern Jordan | Days per week: 0 days 1-3 days 4-7 days vegetables 2.6%, 44.2%, 53.2% | Days per week: 0 days 1-3 days 4-7 days Fruits 3.2%, 51.7 %, 45.2% |  |
| Abudayya et. Al (2009) ^(33)^ | North Gaza Strip Palestine | Vegetables: 30% | Fruits: M: 11.6% F: 16.2% |  |
| Abudayya et. Al (2009) ^(33)^, Abudayya et. Al (2011) ^(34)^ | North Gaza Strip Palestine |  |  | Vegetables and fruits  ≤ 3 t/w 680= 72.96%  > 3 t/w 252= 27.04% |
| AlSabbah et al (2007) ^(50)^ | Palestine | consuming vegetables daily: 45%  West Bank: Vegetables (daily: once or more per day): 43% Gaza: Vegetables (daily: once or more per day): 47% | consuming fruits daily: 31% West Bank: Fruit (daily: once or more per day): 34% Gaza: Fruit (daily: once or more per day): 28% |  |
| Ghrayeb et al (2014) ^(44)^ | Palestine Hebron  governorate, | Servings per day: Vegetable consumption 0 18.2% 1-2 54.4% 3-4 17.6% | Servings per day: Fruit consumption  0 22.4% 1-2 48.1% 3-4 22.1% 5=< 7.4% |  |
| Bashour et. Al (2004) ^(13)^ | the Syrian Arab  Republic | No green vegetables consumption: 50% | fruits 3 times or more daily in the previous week: 11.8% |  |
| Musaiger et al (2014) ^(16)^ | Syria | vegetables  4> 53.7% 4=< 46.3% | Fruits 4> 41.4% 4=< 58.6% |  |
| Darfour-Oduro et al (2018) ^(48)^ | (Syria) 49 LMICs | - |  | - |
| Alzahrani et. Al 2017 ^(69)^, Alzahrani et al (2014) ^(31)^ | Saudi Arabia |  | Fruits less than once daily: 85% |  |
| Alghadir et al (2016) ^(12)^ | Saudi Arabia |  | 4%: did not eat fruit 35%: fruit at least 1–3 times per week 60%: fruit >3 times per week 40%: did not eat fresh fruit and vegetables |  |
| Al-Hazzaa et al (2011) ^(36)^ | three major cities  in Saudi Arabia | Daily vegetables Intake  M: 23.3% F: 22.3% average prevalence of vegetable consumption more than three times per week: 48% | Daily fruits intake M: 16.0% F: 9.6% average prevalence of fruit consumption more than three times per week: 32% |  |
| Al-Hazzaa et al (2013) ^(37)^ | Four cities in  Saudi Arabia  and Britain | Daily intake vegetables 23.0% | Daily intake fruit 13.1% |  |
| Alzahrani et al (2014) ^(31)^, Alzahrani et. Al 2017 ^(68)^ | Saudi Arabia |  | Fruit, Less than once daily 84.6% |  |
| Alsubaie et al (2018) ^(51)^ | Saudi Arabia | 11–12 years Vegetable consumption Daily: 25.9% Not every day 74.1% | 11–12 years Fruit consumption Daily 27.0% Not every day 73.0% |  |
| Aljuaid et al (2020) ^(51)^ | Saudi Arabia | consumption at least once daily vegetable 20.6% | consumption at least once daily fruits: 18.2% |  |
| Aedh et al (2019) ^(29)^ | Saudi Arabia |  |  | Vegetable and fruits times/week >=3 times per week 49.58% <3 times per week 27.92% For the healthy |
| Mahfouz et al (2012) ^(10)^, Mahfouz te al (2011) ^(11)^ | Aseer Region,  southwestern Saudi Arabia | consumption the past 7 days Fresh vegetables, M: consumed 83.1% , didn't: 16.9% F: consumed: 89.9%, didn't: 10.1% | consumption the past 7 days Fresh fruits M: consumed: 72.9%, didn't: 27.1%  F: consumed: 76.6%, didn't: 23.4%  Fresh juices M: consumed: 66.5%, didn't: 33.5% F: consumed: 76.1%, didn't: 23.9% |  |
| Mahfouz et al (2012) ^(10)^, Mahfouz te al (2011) ^(11)^ | Saudi Arabia | Fresh vegetables  M: 3/day:11.1% 2/day: 16.3% once daily: 31.3% 4-5 weekly: 12.6% 1-3 weekly: 11.8% never: 16.9% F: 3/day: 8.9% 2/day: 14.8%  4-5 weekly: 11.5% 1-3 weekly: 12.1% neve: 10.1%  once daily: 42.6% | fresh fruits M: 3/day: 9.0% 2/day: 12.2% once daily: 27.2% 4-5/ week: 8.5% 1-3/ week: 16.0% never: 27.1%  F:  3/ day: 6.1% 2/day: 16.3% once daily: 31.3% 4-5/ week: 6.9% 1-3/ week: 16.0% never: 23.4% fresh juices M:  3/day:7.4% 2/day: 9.1% once daily: 28.4% 4-5/ week: 6.7% 1-3/ week: 14.9% never: 33.5% F: 3/ day: 5.0% 2/day: 14.0% once daily: 33.2% 4-5 weekly: 6.8% 1-3/ weekly: 17.1%  never: 23.9% |  |
| AlBuhairan et al (2015) ^(67)^ | 13 regions  of Saudi Arabia | Vegetable intake (>=1 servings) Total: 54.3% 95%CI: 50.7, 58.0 | Fruit intake (>=1 servings) Total: 38.1% 95%CI: 34.0, 42.1 |  |
| El-Kassas et al (2017) ^(52)^ | North Lebanon | Types of snacks usually consumed  Vegetables: 4.1%  Daily intake of vegetables  Adequate: 43.8%  Inadequate: 56.2% | Types of snacks usually consumed  Fruits: 19.2%   Daily intake of fruits  Adequate: 45.2%  Inadequate: 54.8% |  |
| Hamrani et al (2015) ^(72)^ | the northern part of Morocco Kenitra city | vegetables on a daily basis: 49 %  Fruits: >= 7times/ week proportion: 27.80% 95%CI: 24.07%, 30.79% | fruits on a daily basis: 28 % Fruits: >= 7times/ week proportion: 27.80% 95%CI: 24.07%, 30.79% |  |
| El-Ammari et al (2020) ^(6)^ | North-central  Morocco | - |  |  |
| Amahmid et al (2019) ^(30)^ | in districts with  medium living  standards in Marrakesh (Morocco) | Vegetable intake frequency  <3 times/day: 49.8%  ≥3 times/day 50.2% | Fruit intake frequency Once a day 42.5 % 2–3 days per week 45.8%  < 2 days per week 11.6 % |  |
| Azekour et al (2019) ^(41)^ | Morocco | - |  | - |
| ElAchhab et al (2018) ^(70)^ | Morocco | % 95%CI  Vegetables (< 7 times/wk): M:  66.9%  95%CI: 59.9—73.8 F: 57.5% 95%CI: 50.3—64.1 | Fruits (< 7 times/wk): M:  81.3% 95%CI: 74.9—87.0  F:  79.6% 95%CI: 73.5—85.0 |  |
| Badr et al (2017) ^(47)^ | Kuwait | 1)daily consumption Vegetables: M: 21.8% F: 16.7% 2) Frequencies fruits: no: 41.3%, 1-2 23.0%, 2+: 35.6% | 1)daily consumption fruit:  M: 38.1%  F: 33.2% 2) Frequencies fruits: no: 41.3%, 1-2 23.0%, 2+: 35.6% |  |
| Allafi et al (2014) ^(38)^ | Kuwait | Vegetables: M: (P value) 0.02 Never: 10.5% 1–3: 37.5% 4–6: 26.0% more of equal to 7: 26.0% Vegetables: F:  (P value) 0.02  Never: 10.6% 1–3: 45.0% 4–6: 22.3% more of equal to 7: 22.1% | Fruits  M: (P value) 0.001 Never: 8.7% 1–3: 50.7% 4–6: 23.1% more of equal to 7: 17.5% Fruits: F: (P value) 0.001 Never: 14.3%  1–3: 53.7% 4–6: 20.2% more of equal to 7: 11.8% |  |
| Musaiger et al (2016) ^(17)^ | Sudan | -Vegetables (green leafy, tuber, and roots) <4: M: 33.1% F: 39.5% (p-value <.042) Total: 36.1% 4+: M: 66.9% F: 60.5% Total: 63.9% | -Fruit <4:  M: 72.0% F: 67.6% (p-value <.140) Total: 69.9% 4+:  M: 28.0% F: 32.4% Total:30.1% |  |
| Abbass et al (2019) ^(66)^ | Egypt |  |  | Fruits & Vegetables ≤ 2 times/week 24.18% 3–6 times/week 28.8% 1–6 times/day 47.01% |
| Musaiger et al (2014) ^(46)^ | Iraq | Daily vegetable consumption  M: 46.1%  F: 62.3 | Daily fruit consumption  M: 24.3%  F: 46.3% |  |
